# Comparison of the effectiveness of lauromacrogol injection for ablation and microwave ablation in the treatment of predominantly cystic thyroid nodules: a multicentre study

**DOI:** 10.1186/s12885-023-11301-7

**Published:** 2023-08-23

**Authors:** Xin Min, Zheng Zhang, Yanwei Chen, Shuangshuang Zhao, Jingwen Ge, Huajiao Zhao, Yun Cai, Hui Chen, Jun Shao, Yanfei Jing, Baoding Chen

**Affiliations:** 1 Department of Medical Ultrasound, Affiliated Hospital of Jiangsu University, Zhenjiang, 212000 China; 2 Department of Medical Ultrasound, Changzhou First People’s Hospital and The Third Affiliated Hospital of Soochow University, Changzhou, 213003 China; 3 Department of Medical Ultrasound, The First People’s Hospital of Kunshan Affiliated to Jiangsu University, Kunshan, 215132 China; 4 Department of Medical Ultrasound, The Fifth People’s Hospital of Wuxi, The Medical School of Jiangnan University, Wuxi, 214000 Jiangsu China

**Keywords:** Microwave ablation, Lauromacrogol injection for ablation, Ultrasound medicine, Predominantly cystic thyroid nodules

## Abstract

**Purpose:**

To compare the therapeutic efficacy and safety of microwave ablation (MWA) and lauromacrogol injection for ablation (LIA) for benign predominantly cystic thyroid nodules.

**Materials and methods:**

In this retrospective study, 85 patients with predominantly cystic thyroid nodules (PCTNs) who underwent microwave ablation (MWA) or lauromacrogol injection for ablation (LIA) between June 2019 and August 2022 at three hospitals were included in our research. Forty-six patients were treated with microwave ablation, and thirty-nine patients were treated with lauromacrogol injection for ablation. The baseline characteristics, nodal volume, volume reduction rate (VRR), and incidence of postoperative complications were compared between these two groups.

**Results:**

After treatment, there were significant differences in the thyroid nodule volume and the volume reduction rate (VRR) at different follow-up times between the groups (p < 0.001). There were no significant differences in the nodal volume or the volume reduction rate (VRR) between the MWA group and the LIA group at 1, 3, 6, and 12 months (p > 0.05). Of note, no serious intraoperative or postoperative complications occurred in the corresponding group.

**Conclusion:**

MWA and LIA are very effective and safe strategies for the treatment of predominantly cystic thyroid nodules. However, LIA is more advantageous in that it is less expensive and has a shorter length of hospital stay than MWA.

## Introduction

In recent decades, the advancement of imaging technology, particularly the use of high-resolution ultrasound (US), has improved the diagnosis of thyroid nodules (TNs) [1–3]. According to a large body of evidence, approximately 15–30% of thyroid nodules in adults are diagnosed as cystic or mainly cystic nodules (50% cystic components) [3–6]. Most of these nodules are large and cause a choking sensation when swallowing and a sensation of a foreign body in the throat [7–9]. Due to their rapid growth and large size, the nodules may compress important anatomical structures such as the trachea, oesophagus, and large blood vessels in the neck [8, 10, 11].

Traditionally, surgery is the main treatment for predominantly cystic thyroid nodules (PCTNs) despite its disadvantages such as scarring, nerve damage, and the risk of hypothyroidism [12–14]. In recent years, some minimally invasive approaches, including physical and chemical ablation therapy, have been widely adopted for the treatment of predominantly cystic thyroid nodules [8, 15, 16]. Chemical ablation, with either absolute ethanol or lauromacrogol, is often used for simple cystic thyroid nodules [17, 18]. However, absolute ethanol is well diffused, easily spilled, and difficult to control in ablation. Lauromacrogol (polyoxyethylene lauryl ether) is a sclerosing agent with a local anaesthetic effect. It has been reported that lauromacrogol has barely any effect on many organs with large benign cystic lesions, such as the liver, kidney, and pancreas [19]. Therefore, many clinicians have devoted themselves to exploring the use of lauromacrogol as an alternative to ethanol ablation in the treatment of benign cystic thyroid nodules [20]. In addition to chemical ablation, thermal ablation has been widely used as an effective surgical treatment in clinical practice. It has high feasibility and low complication rate [21]. In recent years, microwave ablation has been shown to be a promising and safe new method for the treatment of thyroid nodules including those with predominantly cystic thyroid nodules [22].

However, there are few studies comparing the efficacy of microwave ablation and lauromacrogol injection for ablation in the treatment of predominantly cystic thyroid nodules. This study aims to compare the efficacy of MWA and LIA in the treatment of predominantly cystic thyroid nodules, and it is hoped that the study will provide more effective information for clinical decision-making and the selection of patients with predominantly cystic thyroid nodules.

## Materials and methods

This study was a retrospective study that was approved by the Ethics Committee of the Affiliated Hospital of Jiangsu University (SWYXLL20190225–2). Informed consent was not required for the review of ultrasound images and medical records. To ensure uniformity among the three study centres, the data were analysed and processed by personnel who strictly followed the inclusion and exclusion criteria of the study. This study followed the Strengthening the Reporting of Observational Studies in Epidemiology (STROBE) reporting guideline for observational studies [23].

### Patients

Before each procedure, all the patients who were scheduled for ultrasound-guided fine-needle aspiration (FNA), MWA or LIA provided written informed consent.

Inclusion criteria: patients who were older than 18 years of age; patients with preoperative ultrasound images showing predominantly cystic thyroid nodules (PCTNs, 50% < cystic component < 90% ); patients with thyroid nodules without signs of malignancy (each nodule underwent at least two fine-needle aspiration cytology (FNAC) examinations to rule out the possibility of malignancy and ultrasound suggests no suspicious ultrasound features such as calcification, poorly defined borders, aspect ratios > 1 and so on); patients with relevant laboratory test results (including routine blood tests, coagulation tests, and thyroid function tests) within the normal range; and patients with thyroid gland disease who had not undergone any previous surgical treatment (including surgical intervention or chemical or physical ablation).

The exclusion criteria were as follows: patients with a malignant tumour confirmed by fine needle aspiration cytology; patients with malignant ultrasound features of thyroid nodules (calcification, poorly defined borders, aspect ratios > 1 and so on); patients with an abnormal coagulation function; and patients with serious primary illnesses such as comorbidities or other serious infectious diseases.

### Group criteria

According to the need of the study, whether it was the MWA group or the LIA group, we divided the patients into two groups based on the size of the thyroid initial volume. Patients with smaller thyroid volume were group A (volume < 10 ml) and patients with larger thyroid volume were group B (volume ≥ 10 ml).

### Preablation evaluation

Before the operation, the patients underwent relevant tests. (1) Laboratory tests included routine blood tests, blood coagulation tests, and thyroid function tests. (2) The pathological results of ultrasound-guided fine needle aspiration cytology were benign. (3) The thyroid nodules were examined via ultrasound, and the location and volume of the nodules, the proportion of cystic components, and the blood flow in and around the nodules were evaluated in detail.

### Ablation procedure

All ablation procedures were performed by sonographers with more than 10 years of experience in performing ultrasound intervention.

The microwave ablation (MWA) procedure: The patient was placed in the supine position with the neck fully exposed. The patient was then routinely disinfected and covered with towels. According to the location of the lesion, 2% lidocaine was used for local infiltration anaesthesia. An 18G puncture needle was placed into the cystic part of the nodule, and as much cystic fluid as possible was extracted. Under the guidance of ultrasound, a 2% lidocaine injection and a 0.9% sodium chloride injection were mixed 1:1, and local infiltration anaesthesia was administered in the space between the anterior thyroid capsule and the anterior cervical muscle group. To prevent causing a thermal burn injury to the normal tissues during the operation, 0.9% sodium chloride was injected in the local gap to form a liquid isolation zone. Then, the microwave ablation needle was inserted into the thyroid nodules according to the predesigned puncture path, and ablation was performed by a continuous moving method, with a power of 30–40 W, from deep to shallow, from the lower pole to the upper pole, layer by layer, until the whole nodule was ablated. If necessary, contrast-enhanced ultrasound should be used to determine the extent of ablation for lesions to prevent residual lesions and additional organ damage.

The Lauromacrogol injection for ablation (LIA) procedure: The patient was positioned supine so that the neck was slightly extended. Routine disinfection and draping were performed before local anaesthesia was administered. Under ultrasound guidance, an 18G puncture needle was inserted into the centre of the cystic dark area, and all the cystic fluid was extracted. A 0.9% sodium chloride injection or a small amount of alcohol or lauromacrogol was used for multiple flushing (the specific volume of liquid to be replaced was calculated according to the volume of the nodule and the proportion of cystic part), and then, lauromacrogol was injected.

### Follow-up and assessments

Postoperative complications such as intraoperative bleeding and organ damage and postoperative complications such as infection and abnormal voice were recorded in a timely manner. Thyroid ultrasound was performed at 1, 3, 6, and 12 months after thyroid treatment and every 6 months thereafter, and contrast-enhanced ultrasound was performed if necessary. The size of the nodules was observed, and the volume of the nodules was measured (V = πabc/6, where a is the maximum diameter, and b and c are the other two perpendicular diameters) [24].The volume reduction rate (VRR) was calculated at different time points. VRR= (initial volume-final volume) ×100/initial volume [25].

### Statistical analysis

Statistical analysis of the data was performed using SPSS (IBM SPSS 26.0). The continuous data were expressed as the means ± standard deviations (Χ ± SD). Independent samples t-tests were used to compare the changes in nodule volume and rate of volume reduction before and after treatment, provided that normality of the data was ensured. The intergroup comparison of counting data was conducted by χ2 test. All P values were two-sided, and P < 0.05 indicated that the difference was statistically significant.

## Results

**Fig. 1:**
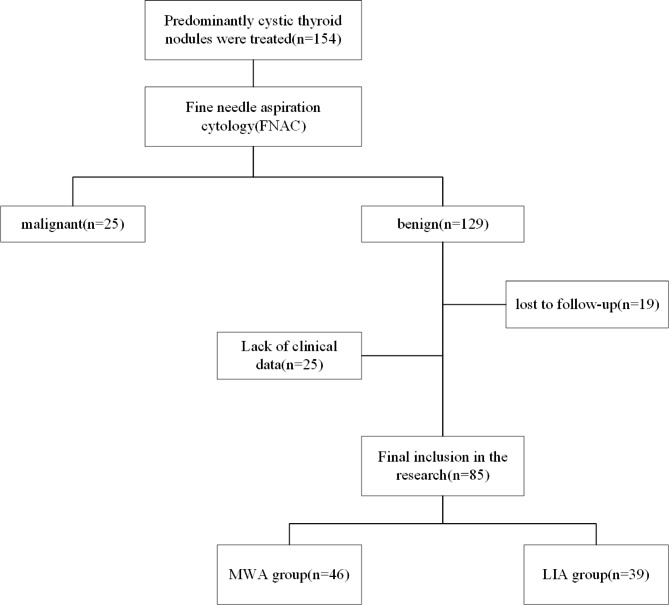
Flowchart of the patient enrollment process

A total of 85 patients participated in our retrospective research, including 46 patients in the MWA group and 39 in the LIA group. After following the strict inclusion criteria, 85 patients with 85 nodules were included in the research (Fig. 1).

### Baseline characteristics of the patients

There were 4 males and 42 females in the MWA group. The youngest patient was 24 years old, the oldest was 79. The mean age was 50.30 ± 14.07 years. In another group, there were 8 males and 31 females. The youngest patient was 24 years old, and the oldest patient was 72 years old. The mean age was 49.79 ± 13.14 years. In the MWA group, the volume of the nodules was 2.67–33.40 ml before treatment, with an average of 11.03 ± 7.16 ml. The volume of nodules in the LIA group before treatment was 2.53–28.33 ml, with an average of 11.41 ± 6.53 ml. Before treatment, there were no significant differences in sex, age, long diameter of nodules, initial volume, or location between the two groups (all P > 0.05). (Table 1)

**Table 1:** Baseline characteristics of the patients with predominant cystic thyroid nodules

	MWA group	LIA group	P value
Patient	46	39	
Sex			0.134
females	42	31	
males	4	8	
Age	50.30 ± 14.07	49.79 ± 13.14	0.864
Nodules	46	39	
Long diameter (mm)	32.03 ± 7.83	35.39 ± 6.85	0.690
Initial Volume (ml)	11.03 ± 7.16	11.41 ± 6.53	0.799
Location			
left	35	11	0.864
right	14	21	
Thyroid function			
TSH	1.68 ± 0.90	2.07 ± 1.05	0.074
TgAb	12.54 ± 1.69	13.29 ± 2.86	0.153
TPOAb	13.16 ± 5.36	12.31 ± 6.29	0.501
Follow-up time (months)	16.65 ± 1.62	17.05 ± 1.43	0.389

Values are presented as the mean ± SD.

TSH, thyroid-stimulating hormone; TgAb, thyroglobulin antibodies; TPOAb, anti-thyroid peroxidase antibody.

### Comparison of the volume and VRR between the two groups

**Fig. 2:**
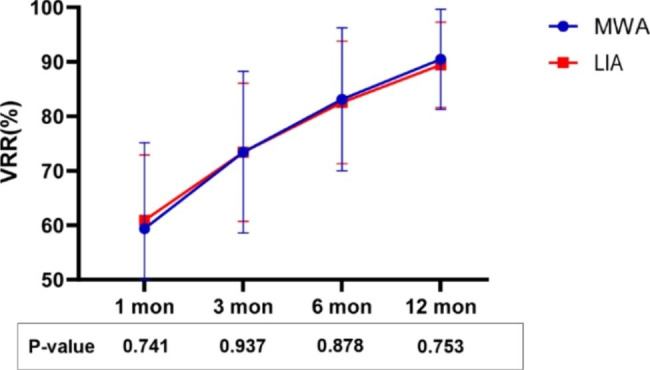
The VRRs of PTNs after MWA or LIA at the postoperative follow-ups. (VRR, volume reduction rate; MWA, microwave ablation; LIA, lauromacrogol injection for ablation; mon, months)

The mean nodal volume in the MWA group before treatment was 11.03 ± 7.16 ml, 2.80 ± 2.31 ml at 6 months and 1.14 ± 1.31 ml at 12 months. The estimated mean VRR at 1, 3, 6, and 12 months was 59.9%, 73.2%, 82.3%, and 88.9%, respectively. In comparison, the mean nodal volume in the LIA group was 11.41 ± 6.53 ml before treatment, 3.15 ± 2.55 ml at 6 months, and 1.23 ± 1.16 ml at the 12-month follow-up. The estimated mean VRRs at 1, 3, 6, and 12 months were 60.9%, 73.4%, 82.5%, and 89.4%, respectively (Fig. 2). In both the MWA group and the LIA group, the nodule volumes at different time points after operation were significantly reduced compared with those before operation (P < 0.001), and postoperative VRR increased significantly with the extension of postoperative follow-up time (P < 0.001). When comparing the reduction in the mean nodule volume after MWA and LIA, no significant difference was found between these two groups (p > 0.05). (Table 2).

**Table 2:** Comparison of the Volume and VRR Between the Two Groups

	MWA group		EA group		P value
	Volume(ml)	VRR(%)	Volume(ml)	VRR(%)	
Baseline	11.03 ± 7.16		11.41 ± 6.53		
1 month after treatment	4.31 ± 3.15	59.9	4.61 ± 3.30	60.9	0.741
3 months after treatment	2.80 ± 2.31	73.2	3.15 ± 2.55	73.4	0.937
6 months after treatment	1.81 ± 1.81	82.3	2.07 ± 1.92	82.5	0.878
12 months after treatment	1.14 ± 1.31	88.9	1.23 ± 1.16	89.4	0.753

MWA, microwave ablation; LIA, lauromacrogol injection for ablation; VRR, volume reduction rate.

### Comparison of treatment effects of thyroid nodules of different sizes

The subgroup study showed that in the MWA group, there was no significant difference between group A and group B in terms of mean VRR at 1 month, 3 months, 6 months and 12 months after ablation (all p > 0.05). Whereas in LIA group, there was no significant difference in VRR between group A and group B at 1 month, 3 months, 6 months and 12 months after ablation (all p > 0.05) (Table 3).

**Table 3:** VRR in different subgroups of thyroid nodules

	MWA group			LIA group		
	group A (n = 23)	Group B (n = 23)	P value	Group A (n = 20)	Group B (n = 19)	P value
VRR at 1 month	61.1	58.7	0.598	58.5	63.4	0.202
VRR at 3 months	74.8	71.5	0.456	70.9	76.0	0.212
VRR at 6 months	83.8	80.8	0.447	81.0	84.1	0.407
VRR at 12 months	89.7	88.2	0.615	89.1	89.8	0.795

MWA, microwave ablation; LIA, lauromacrogol injection for ablation; VRR, volume reduction rate. Group A, patients with small nodules (nodule volume < 10mL); Group B, patients with big nodules (nodule volume ≥ 10mL).

### Comparison of the clinical characteristics between the two groups

The mean number of hospital days in the MWA group was 1.09 ± 0.28, and the average cost of hospitalization was 11.21 ± 0.77 K. The mean number of hospital days in the LIA group was 0.26 ± 0.44, and the average cost of hospitalization was 5.40 ± 0.50 K. In general, there was a significant difference in the length of stay and the cost of hospitalization. (Table 4)

### Safety evaluation

None of the patients had any major complications during or after the therapeutic procedure, such as vocal cord destruction, skin infection or scarring, or damage to the trachea or oesophagus. Three patients in the MWA group experienced minor complications, while 2 patients in the LIA group experienced minor complications; all cases of complications resolved within 3 months. (Table 4)

**Table 4:** Comparison of the clinical characteristics between the two groups

	MWA group	LIA group	P value	
Patient	46	39		
Hospital days	1.09 ± 0.28	0.26 ± 0.44	< 0.001	
Inpatient costs(k)	11.21 ± 0.77	5.40 ± 0.50	< 0.001	
Complication				
Major complication	0	0	/	
Minor complication	3	2	0.789	

Values are presented as the mean ± SD. MWA, microwave ablation; LIA, lauromacrogol injection for ablation.

## Discussion

The rapid development of ultrasound equipment has led to an increasing number of thyroid nodes, mostly benign, being found in daily examinations [2, 26, 27]. For predominantly cystic thyroid nodules, surgery is accepted as the preferred therapeutic option, especially when there is tracheal or oesophageal compression or protrusion from the skin [12, 28]. However, surgery usually has several negative effects, including intraoperative nerve paralysis or injury, postoperative neck haematoma formation, wound infection, and the need for life-long medication therapy after thyroidectomy [29, 30]. In recent years, several minimally invasive methods, such as percutaneous ethanol injection (PEI), lauromacrogol injection for ablation (LIA), microwave ablation (MWA), and radiofrequency ablation (RFA), have been widely used in the treatment of benign thyroid nodules [31–34].

The safety and efficacy of these methods have been previously reported. However, there have been few systematic comparative studies on whether one of these ablation methods is superior to others. Hence, this study is a primary and retrospective analysis comparing MWA and LIA in terms of their effectiveness and safety in the treatment of predominantly cystic thyroid nodules. This study is expected to provide patients with thyroid nodules and clinicals with more information for clinical decision-making.

In recent decades, lauromacrogol sclerotherapy has played an increasingly important role in the treatment of other benign diseases. Lauromacrogol has been previously used in the treatment of gastrointestinal bleeding and haemorrhoids, resulting in good therapeutic effects [35, 36].Further research has proven its great potential in the treatment of cysts. It was first used in the treatment of hepatic cysts [37]. In the past 10 years, several studies have shown that lauromacrogol treatment is more advantageous in terms of efficacy, incidence of complications and cost than traditional liquid sclerotherapy. It has been clinically accepted and used in the treatment of benign cystic thyroid nodules [38]. Lauromacrogol is a liquid sclerosing agent that is injected to treat the relevant disease by producing inflammation and fibrosis in the thyroid tissue [39]. It has also been shown that lauromacrogol can be safely used without causing peri-thyroidal adhesions or altering thyroid function [39]. In the YiJie Dong study [40], 142 benign cystic thyroid nodules in 137 patients were treated with LIA after cytological confirmation of benignancy. At the 12-month postoperative follow-up, the mean size of the thyroid nodules decreased from 18.4 ml to 3.6 ml. Treatment was considered effective with a VRR > 50%, and the treatment success rate was 73.2% (104/142). Another study [41] demonstrated that the volume of 158 cystic or predominantly cystic thyroid nodules in 143 patients were reduced from an initial volume of 15.6 ml to a mean volume of 0.6 ml at the 12-month postoperative follow-up. At the 12-month postoperative follow-up, the volume of all the nodules were reduced by > 70%. Both studies showed that lauromacrogol injection for ablation (LIA) is a safe and effective treatment for predominantly cystic thyroids. Our study had similar findings, with thyroid nodules shrinking from 15 ml before ablation to 12 ml 12 months after the procedure, with a volume reduction rate (VRR) of 75%.

A minimally invasive procedure used for the treatment of benign thyroid nodules is microwave ablation. This novel treatment technique is a safe and effective treatment for benign thyroid nodules and recurrent thyroid cancer. In the Yue et al. study [42]. A total of 474 benign thyroid nodules in 435 patients were treated with ultrasound-guided MWA, and the overall VRR of the thyroid nodules after 6 months of follow-up was 65%, with a VRR of cystic nodules being 83%, which is consistent with the VRR of nodules being 82.3% in our study. This suggests that MWA is safe and effective for the treatment of a predominantly cystic thyroid. Of course, previous studies have also shown that MWA can be used for the treatment of other types of thyroid nodules, such as solid or purely cystic nodules.

In the past, a team studied the factors affecting the effectiveness of the treatment, and they suggested that the initial volume might affect the effectiveness of the treatment [43]. Therefore, we studied initial volume as a criterion for grouping. After our study, we found that there was no statistically significant difference between the VRR of group A and group B in both MWA and LIA groups at 1 month, 3 months, 6 months, and 12 months after ablation (all p > 0.05), which ultimately confirms that the initial volume of the thyroid nodule does not affect the effectiveness of treatment. Our study’s findings were inconsistent with theirs, and we believe that the initial volume of thyroid nodules in the two groups at the time of their study was inconsistent, leading to a bias in their results. We strictly controlled the initial volume of thyroid nodules in both groups in our study.

There is no consensus on the best treatment choices for cystic solid nodules, and guidelines do not give a higher recommendation for LIA or MWA. This study shows that the advantages of LIA are the ease of treatment and the cheap total cost of therapy, but the advantages of MWA are the superior treatment outcomes. LIA is an excellent therapy choice for people who desire to shorten their recovery time or lower their treatment costs. However, some studies have found that due to circumstances such as the nodule’s high parenchymal content or the patient’s high blood supply status, partial resorption or even recurrence of the nodule may occur [40]. As a result, if one desires a better outcome and is willing to tolerate a large amount of therapy and a longer treatment duration, MWA would be an excellent treatment option. Therefore, we need to make better clinical choices based on the patient’s general condition and diagnostic needs.

Our study still had several limitations. (1) This study was a retrospective study with potential selective bias. When selecting subjects for the study, they may have been included or excluded due to human factors, which may have affected the results of the study. Future studies need to collect more patient information and develop more stringent inclusion and exclusion criteria. (2) The relatively small sample size of this study may lead to biased results and requires further validation in a randomized clinical trial with a large sample size. (3) Non-comparative and non-homogeneous statistical analyses are also a limitation, and the findings were confirmed by rigorous experimental grouping in future prospective multicentre studies.

## Conclusion

In our research, both MWA and LIA were therapeutically effective and safe in treating predominantly cystic thyroid nodules. MWA or LIA can effectively reduce the thyroid nodule volume and improve the patients’ clinical symptoms. LIA can shorten the length of hospital stay and reduce the overall cost. Hence, based on the patient’s general condition, we must perform a thorough assessment and select the best course of action.

## Data Availability

The datasets used and/or analysed during the current study are available from the corresponding author on reasonable request.
